# Clonality and distribution of clinical *Ureaplasma* isolates recovered from male patients and infertile couples in China

**DOI:** 10.1371/journal.pone.0183947

**Published:** 2017-08-31

**Authors:** Zhi Ruan, Ting Yang, Xinyan Shi, Yingying Kong, Xinyou Xie, Jun Zhang

**Affiliations:** 1 Clinical Laboratory, Sir Run Run Shaw Hospital, School of Medicine, Zhejiang University, Hangzhou, Zhejiang, China; 2 Biomedical Research Center, Sir Run Run Shaw Hospital, School of Medicine, Zhejiang University, Hangzhou, Zhejiang, China; Miami University, UNITED STATES

## Abstract

*Ureaplasma* spp. have gained increasing recognition as pathogens in both adult and neonatal patients with multiple clinical presentations. However, the clonality of this organism in the male population and infertile couples in China is largely unknown. In this study, 96 (53 *U*. *parvum* and 43 *U*. *urealyticum*) of 103 *Ureaplasma* spp. strains recovered from genital specimens from male patients and 15 pairs of infertile couples were analyzed using multilocus sequence typing (MLST)/expanded multilocus sequence typing (eMLST) schemes. A total of 39 sequence types (STs) and 53 expanded sequence types (eSTs) were identified, with three predominant STs (ST1, ST9 and ST22) and eSTs (eST16, eST41 and eST82). Moreover, phylogenetic analysis revealed two distinct clusters that were highly congruent with the taxonomic differences between the two *Ureaplasma* species. We found significant differences in the distributions of both clusters and sub-groups between the male and female patients (*P <* 0.001). Moreover, 66.7% and 40.0% of the male and female partners of the infertile couples tested positive for *Ureaplasma* spp. The present study also attained excellent agreement of the identification of both *Ureaplasma* species between paired urine and semen specimens from the male partners (k > 0.80). However, this concordance was observed only for the detection of *U*. *urealyticum* within the infertile couples. In conclusion, the distributions of the clusters and sub-groups significantly differed between the male and female patients. *U*. *urealyticum* is more likely to transmit between infertile couples and be associated with clinical manifestations by the specific epidemic clonal lineages.

## Introduction

*Ureaplasma* species are members of class *Mollicutes* and are characterized by lack of a cell wall, a small genome size, and limited biosynthetic abilities [1, 2]. Based on molecular characterization, Ureaplasmas of medical importance are sub-classified into two distinct species (*Ureaplasma parvum* and *Ureaplasma urealyticum*) [3]. Although *Ureaplasma* spp. are regarded as commensal organisms in the urogenital tracts of healthy adults, they are also associated with urogenital tract infections (such as non-gonococcal urethritis, bacterial vaginosis, and prostatitis) [4], adverse pregnancy outcomes (such as chorioamnionitis, preterm birth, spontaneous abortion, and stillbirth) [5, 6], bacteremia with complications in neonates (such as pneumonia, meningitis, abscesses, and chronic lung disease) [7], and recent reports of hyperammonemia in adults [8].

The etiologic role of *Ureaplasma* spp. in male and female infertility is controversial. Some investigations have shown that the presence of *Ureaplasma* spp. has no influence on semen quality [9, 10]. However, other studies have reported that *Ureaplasma* spp. may alter the sperm concentration, motility, morphology and pH, and are ultimately responsible for male infertility [11, 12]. Knox *et al*. demonstrated that *Ureaplasma* spp. can attach to the sperm surface and may act as a reservoir of infection for female partners [13]. Over the past decade, evidence for potentially pathogenic *Ureaplasma* spp. has accumulated, and a recently published systematic review and meta-analysis suggests that *U*. *urealyticum* may contribute to the development of nongonococcal urethritis (NGU) [9, 11, 14–16]. These data reinforced the contentious association between the two different species and infertility, although more research is warranted.

In light of the multilocus sequence typing (MLST) and expanded multilocus sequence typing (eMLST) schemes for *Ureaplasma* spp. have been recently developed [17, 18], only a few research studies focusing on the population structure of this species have been reported [19, 20]. Moreover, the clonality of *Ureaplasma* spp. isolated from male patients is largely unknown and requires further investigation. Therefore, the aims of the present study are to determine the population distribution of *Ureaplasma* spp. in Chinese male patients using MLST and eMLST analyses and to compare the clonality of *Ureaplasma* spp. in paired urine and semen specimens from male versus female partners of infertile couples.

## Material and methods

### Bacterial strains and clinical specimens

All *Ureaplasma* spp. strains included in this study were collected from clinical patients at Sir Run Run Shaw Hospital, School of Medicine, Zhejiang University, from November 2015 to June 2016. A total of 103 *Ureaplasma* spp. clinical strains were immediately cultured from the first void urine or semen specimens of male patients with asthenozoospermia, azoospermia, urethritis, prostatitis, varicocele, or infertility. Moreover, specimens obtained from the urogenital tracts of 15 pairs of infertile couples who visited the Infertility Center were also analyzed in this study. Paired urine and semen specimens were obtained from the male partners, and cervical or vaginal swabs were collected from their female partners, if they had not received any hormone therapies at the same time as their infertility treatments.

The Mycoplasma IST 2 kit (bioMérieux, Marcy l'Etoile, France) was used to isolate *Mycoplasma hominis* and *Ureaplasma* species [21]. One hundred microliters of semen, urine sediment or a cervical/vaginal swab sample was placed in R1 medium and vortexed rapidly. Then, 3 mL of a mixture supplied with the abovementioned kit was added to the R2 medium. The Mycoplasma IST 2 strip was inoculated with the rehydrated R2 medium to separate *M*. *hominis* and *Ureaplasma* spp. and to provide information about antibiotic susceptibility after a 24- to 48-h incubation.

### DNA extraction and sequencing

To prepare the polymerase chain reaction (PCR) template, 0.5 mL of broth cultures of *Ureaplasma* spp. strains were harvested by centrifugation at 12,000×g for 10 min. The cells were re-suspended in 50 μL of lysis buffer (10 mM Tris-HCl, pH 8.0, 50 mM KCl, 2.5 mM MgCl_2_, and 0.5% Tween 20) and proteinase K (10 mg/mL), and the mixture was incubated at 55°C for 1 h. The sample was heated at 95°C for 10 min and centrifuged at 10,000×g for 1 min to remove debris. The supernatant was utilized immediately or stored at -20°C for future use. PCRs were performed according to our previous study, and an overlapping bidirectional sequencing strategy was designed to sequence both strands using the ABI PRISM^®^ 3730 automated sequencer with the BigDye^®^ Terminator technology according to the manufacturer’s recommendations (Applied Biosystems, Foster City, CA) [17]. The sequencing data were trimmed and assembled using the open-source tools Phred/Phrap/Consed on a Linux server workstation with default settings [22, 23].

### Species identification

To distinguish *U*. *parvum* from *U*. *urealyticum*, PCR was conducted with *Ureaplasma* spp.-positive specimens, as previously described [24]. The primers for *U*. *parvum* were UU295-F (5’-GCCAAGAAAACATTTAATCGCT-3’) and UU295-R (5’-CTGATATTGTCCGCTGCTCATT-3’), and the primers for *U*. *urealyticum* were 10_0588-F (5’-AAAGTTAAAGAGTCTTGGTGGA-3’) and 10_0588-R (5’-AATAGGTAATAGCCTCTTTGAT-3’).

### Multilocus sequence typing (MLST) and expanded multilocus sequence typing (eMLST)

For all of the strains included in this study, an MLST scheme was implemented using primers targeting four housekeeping genes (*ftsH*, *rpl22*, *valS*, and *thrS*) as described in our previous study [17]. Moreover, eMLST targeting the four housekeeping genes used for MLST and an additional two putative virulence loci (*ureG* and *mba-np1*) was performed for the same isolates [18].

The Molecular Evolutionary Genetic Analysis software (MEGA 6.0) was used to perform multiple sequence alignments [25]. Numbers were assigned for novel alleles and sequence types (STs)/expanded sequence types (eSTs) according to our in-house designed MLST/eMLST database [17, 18]. Minimum spanning tree (MSTree) analysis is a graph-based clustering method that can generate graphical results from MLST/eMLST profile data. The PHYLOViZ 2.0 program was used to link the allele designations within the STs/eSTs and draw an MSTree [26]. The MSTree was calculated using Prim's algorithm and incorporated with the eBURST algorithm to generate a comprehensive and precise graphical result. The graphical result included different types of lines used to illustrate the number of shared alleles, and the branch lengths reflected the distances between the different types.

### Phylogenetic analysis

A neighbor-joining tree of *Ureaplasma* spp. isolates was constructed with MEGA 6.0 based on the number of nucleotide differences in the concatenated sequences (total of 2,814 bp) of six loci in the eMLST scheme. A total of 1,000 replicates was used for the bootstrapping analysis to establish clonality and to determine the potential relationships between isolates.

### Statistical analysis

IBM SPSS Statistics 19.0 was used to analyze all data in the present study. The association between sub-groups and genders were analyzed using the Chi-square test. Statistical significance was established at 5%. The patient ages are presented as the mean values with standard deviations (SDs).

To assess the agreement between the detection of *U*. *parvum* and *U*. *urealyticum* in the paired urine and semen specimens from infertile men and the species distributions between partners of infertile couples, we used k values (nominal scale variables) as proposed by Landis and Koch [27]. The guidelines for the interpretation of k were as follows: k < 0.20, poor agreement; k = 0.21–0.40, fair agreement; k = 0.41–0.60, moderate agreement; k = 0.61–0.80, substantial agreement; and k = 0.81–1.00, excellent agreement.

The diversity of the MLST/eMLST sequence types was assessed using the Hunter-Gaston Diversity Index [28]. The diversity index (DI) ranges from 0.0 for no diversity to 1.0 for complete diversity. The Hunter-Gaston estimate of diversity incorporated a finite sample adjustment. The results included 95% confidence intervals (CIs), which resulted in precise DI values with upper and lower boundaries.

### Ethics statement

All enrolled patients provided written informed consent prior to data collection. The study and enrollment procedures were approved by the Institutional Ethics Committee of Sir Run Run Shaw Hospital, School of Medicine, Zhejiang University (20150714–1).

## Results

### Genetic lineages of 103 *Ureaplasma* spp. strains recovered from male patients

**Fig 1:**
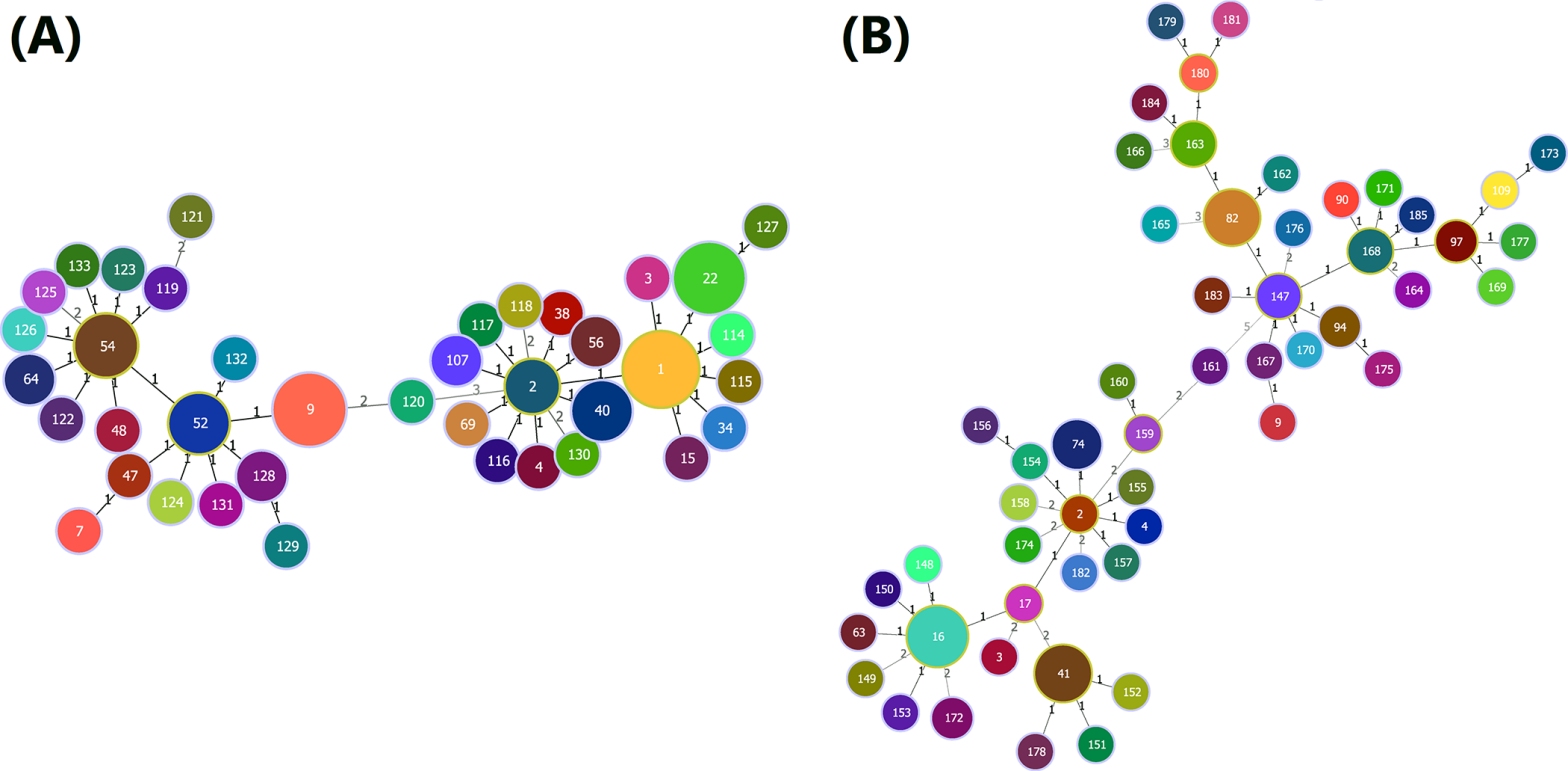
Minimum spanning tree analysis of *Ureaplasma* spp. isolates based on multilocus sequence typing (MLST) and expanded multilocus sequence typing (eMLST) data. Each circle represents an independent sequence type (ST) (panel A) or expanded sequence type (eST) (panel B). The size of each circle corresponds to a different number of isolates, with larger sizes representing higher isolate quantity. The lines connecting the circles indicate the relationship between different STs/eSTs. Different types of lines represent a difference in one allele (solid lines) and two or more alleles (dashed lines). The numbers on the connecting lines illustrate the number of allelic differences.

A total of 103 *Ureaplasma* spp. strains isolated from the genital specimens of male patients were collected for analysis of the major genetic lineages and evolutionary relatedness. Among these strains, 53 (51.5%) specimens were identified as *U*. *parvum*, 43 (41.7%) belonged to *U*. *urealyticum*, and 7 (6.8%) were composed of both *U*. *parvum* and *U*. *urealyticum*. To determine whether certain *Ureaplasma* lineages were multi-clonal, we also explored the ST and eST distributions using MLST and eMLST analysis, respectively, for 96 (53 *U*. *parvum* and 43 *U*. *urealyticum*) out of the 103 *Ureaplasma* spp. strains. As a result, 39 STs and 53 eSTs were identified. ST1, ST9, and ST22 were the predominant STs and were observed in 16, 13, and 12 isolates, respectively. eST16, eST41, and eST82 were the most frequent eSTs and were observed in 14, 10, and 9 isolates, respectively. We also discovered 21 new STs and 39 new eSTs in the present study (Fig 1).

Examination of the Hunter-Gaston Diversity Index indicated moderate diversity among the STs (DI, 0.933; 95% confidence interval [CI], 0.908 to 0.958) and eSTs (DI, 0.957; 95% CI, 0.937 to 0.978), with the greatest individual diversity observed in *mba-np1* (DI, 0.820; 95% CI, 0.783 to 0.857) and the lowest diversity observed in *valS* (DI, 0.560; 95% CI, 0.510 to 0.610). These results indicated a high discriminatory ability for both the MLST and eMLST schema.

### Phylogenetic analyses and a comparison of male and female patients in sub-groups

**Fig 2:**
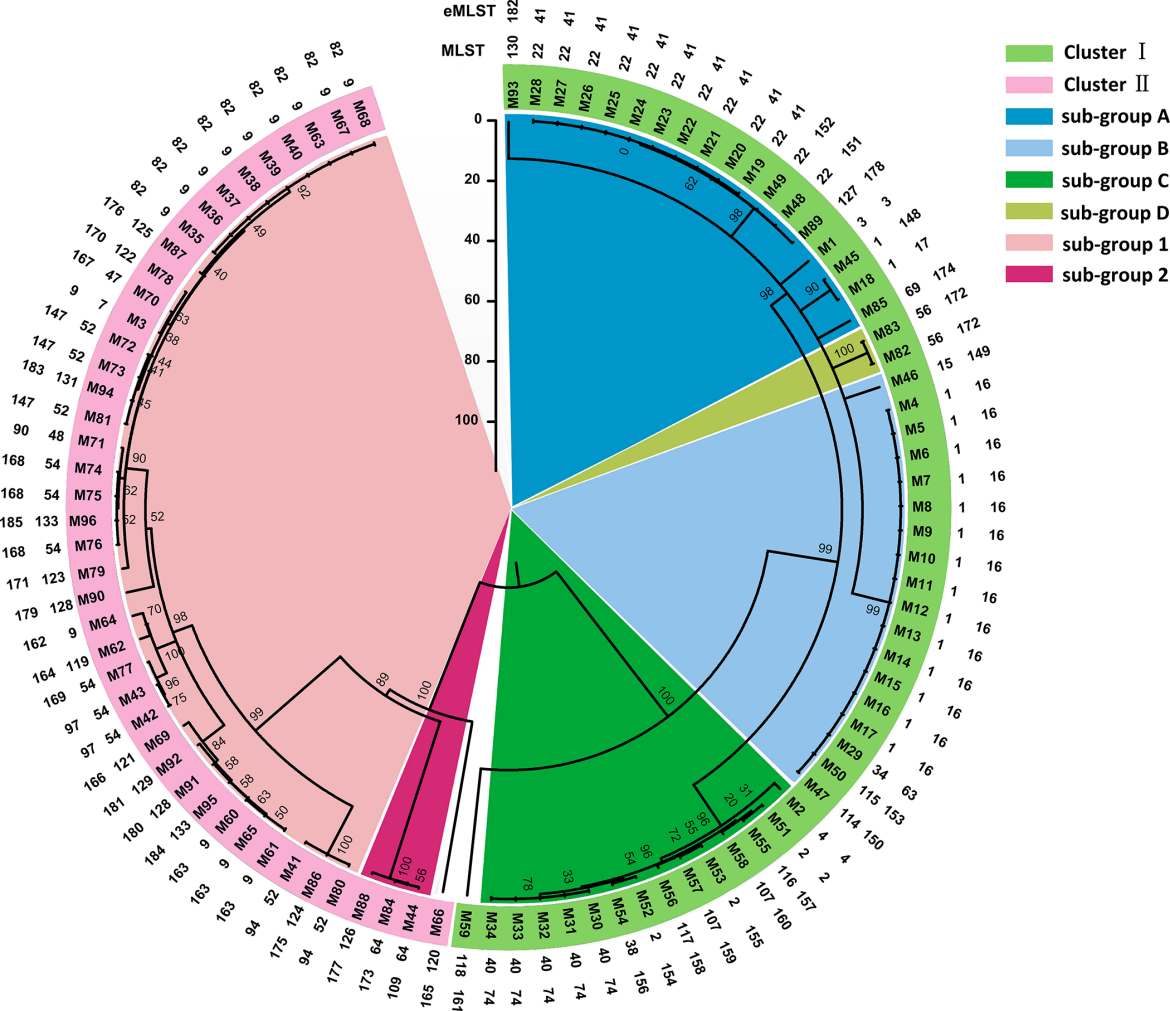
A neighbor-joining tree based on concatenated nucleotide sequences from the six loci was constructed using the MEGA 6.0 software. Two distinct clusters (cluster I and cluster II) were revealed among the 96 strains isolated from the male patients and were highly congruent with the taxonomic differences between the two *Ureaplasma* species. Additionally, four and two sub-groups were found in cluster I and cluster II, respectively. The strains and the corresponding STs and eSTs are given at the tip of each branch. Bootstrap values for 1,000 replicates are indicated on the nodes of the tree.

To investigate the genetic relationships among the 96 *Ureaplasma* spp. strains in the male patients, a neighbor-joining tree was constructed based on the concatenated sequences of six eMLST gene fragments (Fig 2). All 96 strains could be split into two distinct clusters, which were highly congruent with the taxonomic differences between the two *Ureaplasma* species (53 *U*. *parvum* strains in cluster I and 43 *U*. *urealyticum* strains in cluster II). In cluster I, four sub-groups (sub-group A, B, C, and D) were observed. Sub-group A, B, and C comprised the majority of the strains with 18, 18, and 14 strains, respectively. Sub-group D contained only two strains, and the remaining strain was a singleton. Within cluster II, sub-group 1 included 39 isolates, whereas only three strains existed in sub-group 2, and the remaining strain was a singleton. The distributions of the clusters and sub-groups significantly differed between the male and female patients (*P <* 0.001) compared with the data from our previous study [18] (Table 1).

**Table 1:** Comparison of the distribution of sub-groups between male and female patients.

Sex	Cluster I						Cluster II			
	Sub-group A	Sub-group B	Sub-group C	Sub-group D	Sub-group E	Singleton	Sub-group 1	Sub-group 2	Singleton	Sub-total
Male	18	18	14	2	0	1	39	3	1	96
Female ^a^	114	78	30	10	12	1	12	11	1	269
*P* value	*P <* 0.001									

^a^, the data of female patients were analyzed in our previous study (18).

### Distribution of *Ureaplasma* spp. strains in infertile couples

A total of 15 pairs of infertile couples participated in this study. The mean age of the male partners was 32 years (range 25–43 years; SD ± 5.55 years), and the mean age of the female partners was 29 years (range 24–34 years; SD ± 3.34 years). Among the 15 infertile men, 9/15 (60.0%) of the urine specimens and 10/15 (66.7%) of the paired semen specimens were positive for *Ureaplasma* species. Nine men had both urine and paired semen specimens that were positive for the detection of *Ureaplasma* spp., and one male patient had only a positive semen specimen (Table 2). Of the ten *Ureaplasma* spp. strains isolated from the infertile men, five (50.0%) were identified as *U*. *parvum*, four (40.0%) were *U*. *urealyticum*, and one (10%) was characterized as both *U*. *parvum* and *U*. *urealyticum*. Thus, excellent agreement between the paired urine and semen specimens was attained when considering the detection of both *Ureaplasma* species in the infertile men (*U*. *parvum*, k = 0.842; *U*. *urealyticum*, k = 1; and *U*. *parvum* + *U*. *urealyticum*, k = 1) (Table 3).

**Table 2:** *Ureaplasma* species distribution, allele numbers, sequence types (STs), and expanded sequence types (eSTs) in 15 pairs of infertile couples.

Couples		*Ureaplasma* spp. culture	Species	*ftsH*	*rpL22*	*valS*	*thrS*	*ureG*	*mba-np1*	Cluster	Sub-group	ST	eST
Couple 1	Mu-1	Positive	*U*. *urealyticum*	11	3	4	11	5	10	II	2	64	109
	Ms-1	Positive	*U*. *urealyticum*	11	3	4	11	5	10	II	2	64	109
	F1	Positive	*U*. *urealyticum*	11	3	4	11	5	10	II	2	64	109
Couple 2	Mu-2	Positive	*U*. *urealyticum*	5	3	4	5	5	6	II	1	7	9
	Ms-2	Positive	*U*. *urealyticum*	5	3	4	5	5	6	II	1	7	9
	F2	Positive	*U*. *urealyticum*	5	3	4	5	5	6	II	1	7	9
Couple 3	Mu-3	Positive	*U*. *urealyticum*	5	3	4	11	5	6	II	1	48	90
	Ms-3	Positive	*U*. *urealyticum*	5	3	4	11	5	6	II	1	48	90
	F3	Positive	*U*. *urealyticum*	5	3	4	11	5	6	II	1	48	90
Couple 6	Mu-6	Negative	ND	ND	ND	ND	ND	ND	ND	ND	ND	ND	ND
	Ms-6	Negative	ND	ND	ND	ND	ND	ND	ND	ND	ND	ND	ND
	F6	Positive	*U*. *parvum*	1	2	1	1	3	2	I	A	22	41
Couple 8	Mu-8	Positive	*U*. *parvum*	8	1	1	1	1	1	I	D	56	172
	Ms-8	Positive	*U*. *parvum*	8	1	1	1	1	1	I	D	56	172
	F8	Negative	ND	ND	ND	ND	ND	ND	ND	ND	ND	ND	ND
Couple 10	Mu-10	Positive	*U*. *parvum*	1	2	1	1	3	2	I	A	22	41
	Ms-10	Positive	*U*. *parvum*	1	2	1	1	3	2	I	A	22	41
	F10	Positive	*U*. *parvum*	1	2	1	1	3	2	I	A	22	41
Couple 11	Mu-11	Positive	*U*. *parvum*+*U*. *urealyticum*	ND	ND	ND	ND	ND	ND	ND	ND	ND	ND
	Ms-11	Positive	*U*. *parvum*+*U*. *urealyticum*	ND	ND	ND	ND	ND	ND	ND	ND	ND	ND
	F11	Positive	*U*. *parvum*	2	1	1	8	2	2	I	C	40	74
Couple 12	Mu-12	Positive	*U*. *parvum*	53	1	1	1	2	2	I	A	134	186
	Ms-12	Positive	*U*. *parvum*	53	1	1	1	2	2	I	A	134	186
	F12	Negative	ND	ND	ND	ND	ND	ND	ND	ND	ND	ND	ND
Couple 13	Mu-13	Positive	*U*. *parvum*	1	1	1	1	2	1	I	B	1	16
	Ms-13	Positive	*U*. *parvum*	1	1	1	1	2	1	I	B	1	16
	F13	Negative	ND	ND	ND	ND	ND	ND	ND	ND	ND	ND	ND
Couple 14	Mu-14	Negative	ND	ND	ND	ND	ND	ND	ND	ND	ND	ND	ND
	Ms-14	Positive	*U*. *parvum*	1	2	1	1	3	2	I	A	22	41
	F14	Negative	ND	ND	ND	ND	ND	ND	ND	ND	ND	ND	ND
Couple 15	Mu-15	Positive	*U*. *urealyticum*	4	3	4	4	5	6	II	1	9	82
	Ms-15	Positive	*U*. *urealyticum*	4	3	4	4	5	6	II	1	9	82
	F15	Negative	ND	ND	ND	ND	ND	ND	ND	ND	ND	ND	ND
Couple 4,5,7,9	Mu	Negative	ND	ND	ND	ND	ND	ND	ND	ND	ND	ND	ND
	Ms	Negative	ND	ND	ND	ND	ND	ND	ND	ND	ND	ND	ND
	F	Negative	ND	ND	ND	ND	ND	ND	ND	ND	ND	ND	ND

ND, not done; Mu, male urine specimen; Ms, male semen specimen; F, Female specimen.

**Table 3:** Detection of *U*. *parvum* and *U*. *urealyticum* in male and female partners of infertile couples.

Species	Male			Female, n (%)	Urine-semen, k	Male-female, k
	Urine specimens, n (%)	Semen specimens, n (%)	Urine+Semen specimens, n (%)			
*U*. *parvum*	4 (26.7%)	5 (33.3%)	5 (33.3%)	3 (20.0%)	0.842	0
*U*. *urealyticum*	4 (26.7%)	4 (26.7%)	4 (26.7%)	3 (20.0%)	1	0.815
*U*. *parvum*+*U*. *urealyticum*	1 (6.7%)	1 (6.7%)	1 (6.7%)	0 (0)	1	0

k, nominal scale variables.

*Ureaplasma* spp. isolates were detected in 6/15 (40.0%) female partners, with 3/6 (50.0%) and 3/6 (50.0%) identified as *U*. *parvum* and *U*. *urealyticum*, respectively (Table 2). Six couples harbored *Ureaplasma* species in one partner only, including five male members (four identified with *U*. *parvum* and one identified with *U*. *urealyticum*) and one female member (*U*. *parvum*). Notably, the male and female partners of couple 11 displayed different *Ureaplasma* species, with the male partner harboring both *U*. *parvum* and *U*. *urealyticum* and the female partner harboring only *U*. *parvum*. Thus, excellent agreement between the male and female partners was obtained for the detection of *U*. *urealyticum* (k = 0.815), but poor agreement was observed for *U*. *parvum* and the coexistence of *U*. *parvum* and *U*. *urealyticum* (*U*. *parvum*, k = 0; *U*. *parvum* + *U*. *urealyticum*, k = 0) (Table 3).

### Clonality of *Ureaplasma* spp. strains in infertile couples

The MLST analysis revealed eight and five STs among the male and female partners of the 15 pairs of infertile couples (except the male specimens from couple 11), respectively. According to the eMLST data, eight (four eSTs belonging to cluster I and four eSTs belonging to cluster II) and five eSTs (two eSTs belonging to cluster I and three eSTs belonging to cluster II) were identified among the male and female partners of the infertile couples, respectively. All related information is displayed in Table 3.

## Discussion

*Ureaplasma* species have been recognized as important opportunistic pathogens that are usually associated with urogenital infections, adverse pregnancy outcomes, postpartum infections, and hyperammonemia [4–8]. Recently, the differences between *U*. *parvum* and *U*. *urealyticum* in both genome sizes and their correlations with clinical manifestations have received increasing attention. Previous studies indicated that *U*. *urealyticum* might contribute to the development of nongonococcal urethritis (NGU) and the MIC values to most antibiotics tested were significantly higher for *U*. *urealyticum* than for *U*. *parvum* [29]. In the present study, the analysis of 103 *Ureaplasma* spp. strains recovered from male patients showed that the prevalence of *U*. *parvum* was slightly higher than the prevalence of *U*. *urealyticum*.

Analysis of 96 (53 *U*. *parvum* and 43 *U*. *urealyticum*) clinical *Ureaplasma* spp. strains identified a total of 39 STs and 53 eSTs. Both the MLST and eMLST analyses showed high diversity within the clinical *Ureaplasma* spp. isolates studied. As a result, most strains were ST1, ST9, or ST22, whereas eST16, eST41, and eST82 corresponded to the bulk of strains analyzed by the eMLST scheme. As expected, eST16 and eST41, which were incorporated in cluster I and corresponded to ST1 and ST22, respectively, were also observed among the female patients in our previous study [18]. Moreover, eST82, which was incorporated in cluster II and corresponded to ST9, was one of the most predominant sequence types in the male patients but was less commonly observed in the female patients. The predominant sequence types (i.e., ST1, ST9, eST16 and eST41) also corresponded to the known prototype serovars according to the phylogenetic analysis in our previous study [18]. Our data presented here suggest that MLST and eMLST are two valuable tools for the identification of genetic clusters of *Ureaplasma* spp. and can elucidate the relationships between the expected serovars in the absence of actual sequence analysis.

A neighbor-joining tree was constructed to clarify the genetic relationships and clonality among the *Ureaplasma* spp. strains. Two distinct clusters (cluster I and cluster II) were identified within the 96 strains isolated from the male patients. These two clusters were further split into four and two sub-groups, respectively, and the members of each sub-group were closely related. Notably, a high correlation was demonstrated between the clusters and the distributions of two *Ureaplasma* species from cluster I (*U*. *parvum*) to cluster II (*U*. *urealyticum*), which was consistent with previous findings [17–20]. In our previous study, for the 269 strains isolated from female patients, the bulk of *Ureaplasma* spp. strains were split into two subsets: majority cluster I (245 strains, 91.1%) and minority cluster II (24 strains, 8.9%). Regarding to each sub-group, the distributions of sub-group 1 and sub-group 2 were significantly different between the male and female patients. Therefore, we propose that the distributions and genetic dynamics of these two *Ureaplasma* species differ between genders.

Although *Ureaplasma* spp. can be isolated from asymptomatic individuals, some studies suggest that these microorganisms, especially *U*. *urealyticum* are potentially pathogenic, and are often associated with clinical manifestations, including infertility. A dual energy metabolism-dependent effect of *U*. *urealyticum* infection on sperm activity has been discovered, with the inhibition of sperm motility at a low pH and an increase in sperm velocity at a high pH. In *in vitro* fertilization systems, the occurrence of *U*. *urealyticum* in either semen or the female genital tract might have deleterious effects on sperm DNA and result in a decreased pregnancy rate per embryo transfer [30]. In this study, 66.7% and 40.0% of the male and female partners of the infertile couples tested positive for *Ureaplasma* spp., and the distribution of *U*. *parvum* was similar to that of *U*. *urealyticum* in both the male and female partners. This observation was in accordance with the data presented by Zhang et al. in infertile male patients in China, which also showed that *U*. *parvum* tended to be more commonly isolated than *U*. *urealyticum* from fertile men, although *U*. *urealyticum* was more likely to cause male infertility [15]. However, different studies in different patient populations (geographical and ethnic) provide various results concerning which *Ureaplasma* species may be potentially pathogenic. One study reported that the frequency of *U*. *urealyticum* (15%) was higher than that of *U*. *parvum* (4.2%), which was discordant from another study stated that *U*. *parvum* was more prevalent in specimens from infertile men. Additionally, no obvious correlation between the presence of *U*. *urealyticum* and alterations in semen has been reported, although other studies have demonstrated a decrease in the sperm concentration, motility and morphology [9, 11, 14].

Excellent agreement regarding the detection of both *Ureaplasma* species was observed between the paired urine and semen specimens collected from the infertile men, which was consistent with the findings of previous studies [9, 14]. The present study also found excellent agreement between the detection of *U*. *urealyticum* in male and female partners of infertile couples, whereas poor agreement was found for the detection of *U*. *parvum* and the coexistence of *U*. *parvum* and *U*. *urealyticum*. These observations could be explained by a previous finding that *Ureaplasma* spp. were able to attach to the surface of sperm and were not always removed from semen by the standard assisted reproductive technology (ART) washing procedure. They can remain adhered to the surface of spermatozoa, and may act as a reservoir of infection for female partners [13]. Together with the data provided by the previous two studies, our observations suggested that *U*. *parvum* was more likely to be a natural inhabitant of the human urogenital tract and that *U*. *urealyticum* was prone to transmit between infertile couples and be associate with specific hyper-epidemic clonal lineages. Genotyping of *Ureaplasma* spp. isolates could become a powerful tool in the judicial arena for substantiating allegations of sexual assault because almost every couple had a conserved but almost unique ST (only ST22/eST41 was represented more than once). Although this study has extended our knowledge of *Ureaplasma* infections, it has some limitations. The total number of patients included in this study was relatively small. Additionally, continuous surveillance of the clonality and distribution of clinical *Ureaplasma* isolates recovered from infertile couples is warranted to further validate the pathogenic potential of this species.

In conclusion, this study extends previous knowledge regarding the clonality and distribution of clinical *Ureaplasma* isolates recovered from male patients and infertile couples in China. The distributions of *Ureaplasma* species both in the two clusters and in the sub-groups significantly differed between the male and female patients. *U*. *urealyticum* is more likely to transmit between infertile couples and be associated with clinical manifestations by specific epidemic clonal lineages.
